# Patient experiences of the pandemic; exploring the effect of COVID-19 on patients detained under the Mental Health Act

**DOI:** 10.1192/bjo.2021.793

**Published:** 2021-06-18

**Authors:** Emily Watson, Samuel Rowles

**Affiliations:** St Andrews Healthcare

## Abstract

**Aims:**

The current pandemic and the restrictions on liberty that it has necessitated has had a huge impact on society as a whole. We were interested to learn how the constraints of sequential lockdowns and social distancing measures had affected inpatients in a mental health setting, many of whom were already contending with significant restrictions on their freedom.

**Method:**

We conducted structured interviews with 24 service users across the Low Secure and Locked Rehabilitation Division at St Andrews Healthcare Northampton. We interviewed male and female inpatients with diverse diagnoses including emotionally unstable personality disorder, anorexia nervosa, schizophrenia and offending behaviours. All participants were detained under the Mental Health Act throughout the pandemic. Service users were asked the following questions:

- How has the pandemic affected your mood?
- How has it affected your relationship with your family?
- How has it affected your treatment?
- How has the pandemic affected your leave?
- How has it affected how you use your free time?
- Are there any other ways the pandemic has affected you?
- We performed thematic analysis to identify ways the pandemic has affected service users.

**Result:**

Four major themes were identified:

1. Mental health
  Participants reported a decline in mood.
2. Changing relationships

Service users reported that relationships with loved ones in the community had suffered from lack of contact and missing significant life events, however several participants felt that their relationships with peers had strengthened.

3) Delivery of care

Responses were split on the increased reliance on technology to replace face-to-face interaction between patients and team members, with some respondents reporting this as 'less intimidating', while others found this ‘isolating’. Respondents reported reduced contact with MDT members and delays to recovery and step-down placements due to decreased leave.

4) Routine

Respondents reported an increase in free time throughout the pandemic. Some used this to develop hobbies whereas others reported becoming ‘lazy’ and expressed disappointment with the lack of exercise provision.

**Conclusion:**

The pandemic has had significant emotional and psychological effects on society as a whole, but perhaps no group has been more affected than detained patients who have had their lives restricted to a massive degree. This group has been largely marginalised by government guidelines which often fail to consider individuals living in large group settings. By learning from the experiences of these service users we can adapt our practices to alleviate these issues in any future lockdowns and ensure our practices are the least restrictive possible.

